# Childhood bullying and body dissatisfaction in adolescents from marginalised groups

**DOI:** 10.1186/s40337-026-01709-4

**Published:** 2026-07-22

**Authors:** Marie-Christine Opitz, Nora Trompeter, Ulrike Schmidt, Nadia Micali, Helen Sharpe

**Affiliations:** 1 Department of Clinical Psychology, School of Health in Social Sciences, University of Edinburgh, Edinburgh, UK; 2 Great Ormond Street Institute of Child Health, UCL, London, UK; 3 Department of Psychological Medicine, Institute of Psychiatry, Psychology and Neuroscience, King’s College London, London, UK; 4 Center for Eating and Feeding Disorders Research, Mental Health Center Ballerup, Copenhagen University Hospital-Mental Health Services CPH, Copenhagen, Denmark

**Keywords:** Bullying, Peer victimisation, Body dissatisfaction, Adolescence, ALSPAC, Millennium cohort study

## Abstract

**Background:**

Experiences of being bullied have consistently been linked to body dissatisfaction. The present study aimed to investigate the longitudinal relationship between childhood bullying and body image outcomes in adolescents from two large UK-based cohorts. Analyses were conducted separately for marginalised groups to investigate unique risks.

**Methods:**

Data from the Avon Longitudinal Study of Parents and Children (ALSPAC, “Children of the 90s” born in 1991–1993) and the Millennium Cohort Study (MCS, including young people born in 2000–2002) were used. Childhood bullying was reported by *N* = 10,005 children at ages 5–11 (ALSPAC) and *N* = 10,950 children at 7–11 (MSC), while body dissatisfaction was self-reported at ages 10 and 14 (ALSPAC) and 11 and 14 (MSC).

**Results:**

Experiences of childhood bullying were consistently associated with poorer body image outcomes in adolescents. While the ALSPAC cohort showed primarily significant associations for girls, MCS data indicated significant associations for both girls and boys. Marginalised identities did not moderate effects, except for ethnicity in 14-year-old girls (MSC), indicating a weaker effect for ethnic minority girls.

**Conclusions:**

The present study provides further evidence for the role of childhood bullying experiences as a risk factor for body dissatisfaction in adolescents. Further research needs to investigate the role of intersectionality when exploring marginalised identities, as well as the unique role of appearance-based bullying on body dissatisfaction.

**Supplementary Information:**

The online version contains supplementary material available at https://doi.org/10.1186/s40337-026-01709-4.

## Abstract (plain language summary)

The present study explored whether being bullied as a child affects how young people feel about their bodies. Data from two large UK studies (“Children of the 90s” and the “Millennium Cohort Study”) were used, as they provide information across childhood and adolescence. The results show that children who were bullied during childhood were more likely to feel unhappy with their bodies during adolescence. For the “Children of the 90s” study this association was mainly seen in girls. In the ”Millennium Cohort Study”, being bullied was linked to poorer body image in both girls and boys. Additionally, the study looked at whether the effects of bullying were different for children from marginalised groups. Overall, considering young people’s marginalised identity status did not change the link between bullying and body dissatisfaction, with one exception: among 14-year-old girls, the link was slightly weaker for those from ethnic minority backgrounds. Overall, this study adds strong evidence that being bullied in childhood can harm body image later on. Findings further suggest that future research should look more closely at how different aspects of marginalised identities overlap, and whether being bullied specifically in regards to appearance has a particularly strong impact on body dissatisfaction.

**Supplementary Information:**

The online version contains supplementary material available at https://doi.org/10.1186/s40337-026-01709-4.

## Background

Being bullied in childhood has consistently been linked with body dissatisfaction in adolescents, with impacts lasting into adulthood [1–3]. Bullying refers to repeated, intentional acts of interpersonal aggression with an imbalance of power [4]. This includes verbal taunting, physical harm, and social exclusion both in-person and online [5]. Around 30–40% of youth experience bullying [6], with even higher rates in marginalised groups [7]. Marginalised youth might be uniquely affected by bullying, as they might face greater consequences of victimisation due to their target on socially stigmatised identity or appearance (e.g., sexual minority, weight status, rurality or ethnicity) [8–10]. Despite known links between bullying victimisation (being bullied) and body dissatisfaction, little is known about this relationship within specific groups of minority youth.

Links between being bullied and body dissatisfaction in marginalised youth might be explained through both self-stigma theory and social comparison. Self-stigma theory proposes that exposure to public stigma and stereotypes leads individuals to internalise these beliefs and develop self-stigma [11, 12]. Social comparison theory [13] proposes that individuals compare themselves to their peers due to a need for self-evaluation. Such comparisons may be upward (i.e., others are viewed more positively as oneself), downward (i.e., others are viewed more negatively as oneself), or lateral (i.e., others are viewed equal to oneself). Frequent social comparisons, especially upward comparisons, have been linked to more negative body image and disordered eating [14]. In this context, bullying victimisation may lead marginalised youth to internalise negative beliefs about their appearance and make negative comparisons to their peers from majority identities. For instance, qualitative ethnographic work has shown that experiences of ‘misfitting’ in social interactions due to certain behaviours or appearances, is conceptualised as at the core of bullying behaviours, while being closely tied to social comparisons toward a majority culture [15].

Indeed, being bullied has been consistently linked to lower self-esteem and body dissatisfaction [16, 17]. Additionally, findings from qualitative research show that adolescents frequently report reasons for being bullied as relating to their appearance or identity [18]. Importantly, marginalised identities may contribute to negative social comparisons and social stigma regardless of their visibility. That is, stigma and bullying occur both based on physical characteristics (e.g., skin colour, weight status) and social factors (e.g., sexual identity, financial deprivation). However, research within specific groups of marginalised youth is lacking and longitudinal evidence is particularly scarce.

Notably, there is mixed evidence on body dissatisfaction within marginalised groups, and it is further unclear if and how being bullied contributes to this. For example, both male and female US-based adolescents from ethnic minority backgrounds have been shown to experience body image dissatisfaction, however, longitudinal research indicates potential gender differences in how body image dissatisfaction presents across time [19]. Moreover, sexual minority status has consistently been linked to worse body image in men, but not in women [20, 21]. However, no such research exists in adolescents. In adults, relative deprivation has further been shown to be related to body dissatisfaction in both men and women, even though an effect for ‘feeling larger than desired’ was only identified in women from deprived backgrounds [22]. Meanwhile, early childhood socioeconomic deprivation has been longitudinally linked to disordered eating, weight and shape concerns, as well as body dissatisfaction in adolescents [23]. Finally, while more targeted and UK-based research on rural-urban differences in body dissatisfaction is currently still missing, preliminary findings indicate that industrial, semi-industrial, and rural societies might differ in how people’s attractiveness is culturally established, which appears to be primarily linked to different BMI values, i.e., lower BMIs are seen as more attractive in industrialised societies [24].

Understanding links between bullying victimisation and body dissatisfaction among youth, especially marginalised youth, is critical for informing prevention and early intervention. Most anti-bullying programs rely on universal approaches [25], which could be supplemented with targeted support for marginalised youth. To address these gaps, the current study had two main aims. First, to examine whether being bullied at childhood predicts subsequent body image concerns in girls and boys at age 10/11 and age 14, drawing on data from two population-based cohort studies in the UK (ALSPAC, MCS). Second, this study aimed to explore if the association between childhood bullying and subsequent body image concerns is moderated by participants’ weight status, sexual minority status, ethnicity, urbanicity, and/or deprivation status.

## Methods

### Participants and design

The study draws on data from two distinct cohort studies to investigate experiences of different marginalised groups in the UK. Below, we describe the methods for each cohort and how each data source was used for the current study.

*ALSPAC* The ALSPAC birth cohort [26, 27] is a longitudinal study investigating environmental and genetic factors affecting cohort members’ health and development. Participants were children of mothers residing in Avon, UK, with expected delivery between 1st April 1991 and 31st December 1992. 14,541 pregnancies were enrolled, of which 13,988 children were alive at 1 year of age. From these 14,541 initial pregnancies, 338 were from women who had already enrolled with a previous pregnancy, resulting in 14,203 unique mothers who were initially enrolled in the study. The total sample size, including a bolster sample recruited when the oldest children were around 7 years of age, was 15,447 pregnancies. Of these 14,901 children were alive at 1 year of age. Starting during the first trimester of pregnancy parents repeatedly completed questionnaires about both themselves and the study child. Study children were invited to attend annual assessment clinics, which included objective measurements and face-to face interviews as well as separate self-report questionnaires at home from age 7. Ethical approval for the study was obtained from the ALSPAC Ethics and Law Committee and the Local Research Ethics Committees. Participants gave their informed consent for the use of all data collected, in accordance with the guidelines set out by the ALSPAC Ethics and Law Committee at the time. Participants can contact the study team at any time to retrospectively withdraw consent for their data to be used. Study participation is voluntary and during all data collection sweeps, information was provided on the intended use of data. Further detail on the study recruitment is available here: http://www.bristol.ac.uk/alspac/researchers/cohort-profile/. For the current study, we included participants with relevant data on childhood bullying and sex at birth, using only the first registered twin (*n* = 10,005).

*MCS* The Millennium Cohort Study (MCS) is a longitudinal birth cohort led by the Centre for Longitudinal Studies [28]. At the first assessment point in 2001, the study included 18,818 children and 18,552 families from across the UK (England, Scotland, Wales, Northern Ireland). Since then, eight data sweeps have been collected to explore cohort members’ physical, socio-emotional, cognitive, and behavioural development over time. Children living in disadvantaged areas, children of ethnic minority backgrounds, and children growing up in smaller nations of the UK were purposefully over-sampled. Ethical approval for the study was granted by the respective National Health Service (NHS) Research Ethics Committees and the Health and Social Care Business Services Organisation in Northern Ireland [29]. Data from the MCS sweeps were accessed through the UK Data Service (http://www.ukdataservice.ac.uk/).

### Measures

#### Childhood bullying

*ALSPAC*. To classify childhood bullying we used a composite measure available in the ALSPAC cohort that combines information from mother-report and child-report during ages 5–10 from various questionnaires (see details [30]). The measure includes different types of bullying, including both overt and relational bullying (e.g., *Hit/beaten up; Told lies about)*. Children were classified as having experienced childhood bullying if any one item was endorsed as *at least weekly*/*all the time*/*most days*.

*MCS.* Childhood bullying was assessed through an aggregated dichotomous variable, representing the presence of either parent- or child-reported bullying (at least *many times/certainly true/all of the time/most days/about once a week*) at age 7 or age 11 (reference to the past six months) to align the assessment of bullying experiences with the composite measure available in ALSPAC. Five items were included within the analysis, which inquired about the presence and frequency of bullying (including “being picked on and hurt on purpose”) at both ages (see supplementary material for an overview).

#### Body dissatisfaction

*ALSPAC.* Body dissatisfaction at age 10 was assessed using Stunkard figure rating scales [31]. The Stunkard figures are illustrations of different body types ranging from endomorph to ectomorph. Participants were asked to select both their perceived and desired body shape from the figures. A body dissatisfaction score was generated by taking the absolute value of the difference between the perceived and desired body shapes (i.e., higher scores reflecting greater body dissatisfaction). Scores were dichotomised to reflect *no body dissatisfaction* (score of 0) or *some level of body dissatisfaction* (score ≥ 1). Body dissatisfaction at age 14 was assessed using the Body Dissatisfaction Scale [32]. Young people were asked to rate their satisfaction with individual body parts using a 5-point scale from “extremely satisfied” to “extremely dissatisfied”. Based on gender-specific items (“breasts” for girls and “built” for boys), two separate total scale scores were calculated for boys and girls. The internal consistency of this scale was high for both girls (α = 0.93) and boys (α = 0.90). A continuous score was used with high scores indicating high body dissatisfaction.

*MCS.* Appearance Satisfaction was measured with a single item at age 11 and age 14 (“On a scale of 1 to 7 where ‘1’ means completely happy and ‘7’ means not at all happy, how do you feel about the following parts of your life? How do you feel about the way you look?”). The utility of this item has previously been evaluated [33], indicating an adequate to good validity for the single-item assessment in non-clinical populations.

#### Sex

*ALSPAC.* Biological sex of the study child was reported at birth as either *male* or *female*.

*MCS.* Biological sex, rather than gender, was assessed as a binary variable (reported as “male”/”boy” versus “female”/”girl”) at multiple timepoints. For the present study, information on cohort children’s sex was extracted at age 14 and, if missing, substituted from previous timepoints (age 11 or age 7).

#### Sexual orientation

*ALSPAC.* Sexual orientation identity was assessed at age 16 using a self-report question (“Please choose the description that best fits how you think about yourself.”) Response options were: (1) 100% heterosexual; (2) mostly heterosexual, but also attracted to own sex; (3) bisexual; (4) mostly homosexual, but also attracted to opposite sex; (5) 100% homosexual; (6) not sexually attracted to either sex; (7) not sure; (8) other. Responses of “not sure” and “other” were coded as missing. Responses were dichotomised as heterosexual (i.e (1), 100% heterosexual) or sexual minority (i.e., (2–6 – as above).

*MCS.* Sexual minority status was self-reported by young people at age 17. Due to small cell counts, a dichotomous variable was created to contrast being completely heterosexual with minority sexual orientation (“I have felt sexually attracted to…” “More often to < opposite sex>, and at least once to a < same sex>”/ ”About equally often to < opposite sex > and to < opposite sex>”/ “More often to < same sex>, and at least once to a < opposite sex>”/ “Only ever to < same sex>, never to < opposite sex>”/ “I have never felt sexually attracted to anyone at all”).

#### Ethnicity

*ALSPAC.* Ethnicity was coded as *White* vs. *minority* due to small sample sizes for individual ethnic minorities. Information on ethnicity was based on mother-reported data (“What would you consider to be the cultural background of your study child?”) at age 11. For those with missing data, parental ethnicity was used. If either parent reported their ethnicity as non-White, participants were coded as *minority*. If both parents reported their ethnicity as White, participants were coded as *White*. Within the ethnic minority group, most participants reported mixed ethnicity (*n* = 199, 53.21%).

*MCS.* Ethnicity was coded as *White* vs. *minority* (“Mixed”, “Indian”, “Pakistani and Bangladeshi”, “Black or Black British”, “Other Ethnic group (incl. Chinese)”) based on child-reported data at age 11. For missing information, information from a previous wave (i.e., age 7) was used.

#### Weight status

*ALSPAC.* Weight status was coded as *low to average* vs. *higher weight* based on body mass index (BMI), which was calculated using height (m) and weight (kg) measured at a clinic session when the children were aged 10 years. BMI scores were converted to age- and sex-specific BMI z-scores and categories of ‘underweight’ and ‘normal weight’ (i.e., *low to average weight)*, or ‘overweight’ and ‘obese’ (i.e., *higher weight)* using UK references [34].

*MCS.* Similar to the ALSPAC assessment, weight status was coded dichotomously as *low to average weight* or *higher weight* based on the adjusted BMI categories available in the MCS dataset. To create this variable, children’s height and weight were measured at age 11 by the MCS research team and classified according to the UK90 [35].

#### Deprivation status

*ALSPAC.* Neighbourhood deprivation was measured by the Townsend deprivation score, which was ascertained through participant postcodes [36]. The Townsend deprivation score is a census-based index of material deprivation calculated based on car ownership, overcrowded household, owner-occupied household and unemployment in each geographical area. For the current study, we used information from mother-reported postcodes reported at age 11, or age 10 if age 11 information was missing. Deprivation status was coded as *least deprived* (scores 1–4) vs. *more deprived* (score 5) based on the Townsend deprivation quintiles.

*MCS.* Country-specific indicators of (multiple) deprivation are available as deciles within the MCS. In line with previous approaches [37], five categories were created to rank areas from the 20% least deprived to the 20% more deprived areas.

#### Rurality

*MCS.* Postcodes from participants in the MCS are classified into different country-specific types of rural and urban areas based on the Office for National Statistics census data. For the present study, a dichotomous variable was created according to geographical locations for each country to contrast rural and urban areas.

### Data analysis plan

To examine the relationship between childhood bullying and body dissatisfaction in minority youth, we conducted separate stratified regression analyses by sex and minority status (i.e., sexual orientation, ethnicity, weight status, deprivation, rurality), controlling for all other stratifying variables. For each stratified analyses, only participants with complete data on the relevant stratifying variable were included. In line with the analysis protocol published on the Open Science Framework (OSF, https://doi.org/10.17605/OSF.IO/Z7AVS), the analytic sample of the MCS included all participants who had (1) at least one assessment of bullying experiences provided between the ages 7 to 11, and (2) at least one report of body image (age 11 and/or age 14). For twin pairs, one twin was randomly chosen to be included in all analyses. While the protocol stated only significant interaction terms would be further tested as stratified models, all findings from stratified analyses were reported here to align approaches across cohorts. Missing data on outcome variables was imputed across cohorts. Below, we outline the cohort-specific analyses.

*ALSPAC.* Missing data on outcomes were not completely at random (Little MCAR’s test: *X*^2^(2) = 214.07, *p* < .001), but could be explained by observed data and was therefore considered missing at random. Specifically, missingness on body dissatisfaction at age 10 was significantly related to BMI (*r* = .08, *p* < .001), and parental income at age 7 (*r* = − .02, *p* = .048). Missingness on body dissatisfaction at age 14 was significantly related to ethnicity (*r* = .02, *p* = .029), deprivation (*r* = .09, *p* = < .001), sex (*r* = .15, *p* < .001), and parental incomes across childhood (*r* values = − .11 to − .13, *p value*s < .001). However, effect sizes were small. Using the whole sample, missing data on outcomes were imputed through multiple imputations with chained equations (MICE) based on 40 imputations. For the imputation model, all predictor and outcome variables were used. Stratifying variables and parental income at ages 2, 4, 7, and 8 were included as auxiliary variables. Missing data ranged from .01% for age 10 body dissatisfaction to 44.8% for age 14 body dissatisfaction. Imputed datasets were assessed using the fraction of missing information (FMI), which quantifies the portion of total sampling variance attributable to missing data and serves as a suitable indicator for imputations when missingness is extensive [38]. Largest FMI on the imputed models ranged between .01 and .47. Regression models were used to examine the association between childhood bullying and body dissatisfaction at ages 10 and 14 for each stratified sample. Analyses were conducted in Stata version 19. Unstandardised coefficients are reported for all analyses.

*MCS.* Little MCAR’s test on relevant variables in the MCS indicated missingness was not completely at random (*X*^2^(312) = 6094, *p* < .001). Missingness on the body image item at age 11 was significantly related to children’s sex (*r* =  − .03, *p* < .001), children’s ethnicity (*r* = .05, *p* < .001), children’s weight status (*r* = .02, *p* < .05), families’ deprivation status (*r* = − .06. *p* < .001), their rural location (*r* =  − .08, *p* < .001), and children’s experiences of childhood bullying (*r* = .13, *p* < .001). Missingness on the body image item at age 14 was associated with sex (*r* =  − .04, *p* < .001), ethnicity (*r* =  − .06, *p* < .001), weight status (*r* = .02, *p* < .05), sexuality (*r* =  − .03, *p* < .05), families’ deprivation status (*r* =  − .03, *p* < .01), rural location (*r* = .51, *p* < .001), and children’s childhood bullying experiences (*r* = .05, *p* < .001). However, effect sizes were small, except for rurality. As far as possible, missing data on predictors was substituted by information from previous waves (sex, ethnicity, categorical weight status). MICE with 40 imputations and predictors as auxiliary variables was used to impute missing data on outcome variables within the whole sample. Missing data ranged from 3.54% (ethnicity) to 28.7% (sexuality). Pre-processing of MCS data was conducted using R_Studio_, while regression models investigating the association between childhood bullying and body dissatisfaction were conducted in Stata version 18 to align the analysis approach across cohorts (instead of just R_Studio_, as outlined in the protocol).

## Results

### Sample characteristics

**Fig. 1:**
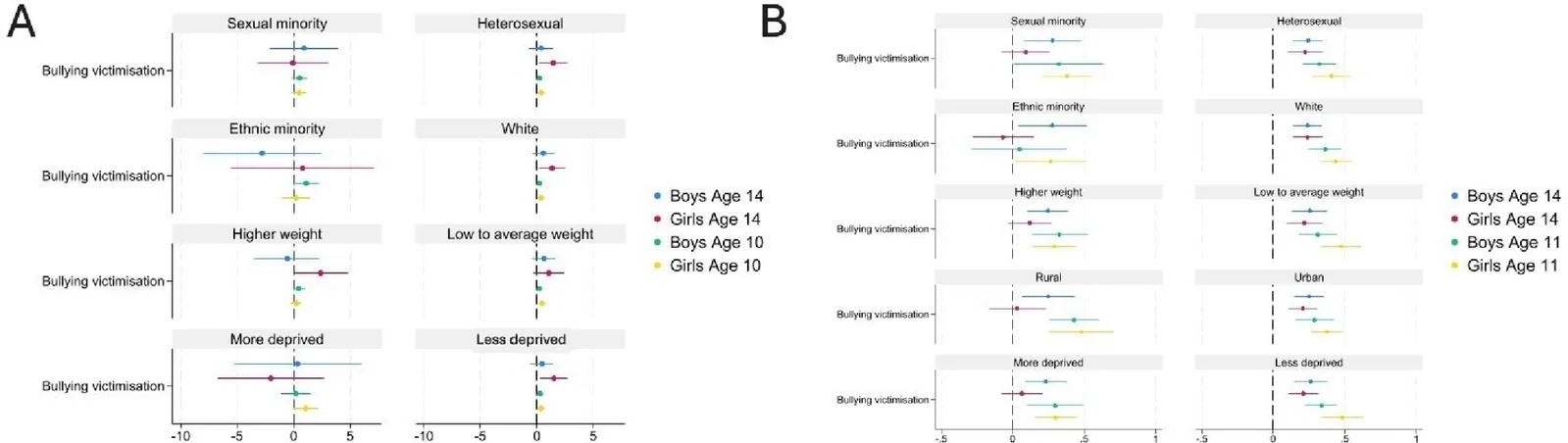
Summary of results by cohort. (**A**) ALSPAC results, (**B**) MCS results. Results from ALSPAC are based on un-standardised estimates. MCS are based on standardised results

Full descriptives for both the ALSPAC and MCS participants are shown in Table 1. In short, the ALSPAC participants were primarily from White, non-deprived families, whereas MCS participants were more diverse. A full overview of all findings from both cohorts is included in the supplementary material (A & B) and graphically illustrated in Fig. 1.

**Table 1:** Sample characteristics by cohort and by sex

	ALSPAC		MCS	
	Boys	Girls	Boys	Girls
*Childhood bullying*				
No	4,029	4,227	3,740	4,096
	78.91%	86.28%	68.67%	74.42%
Yes	1,077	672	1,706	1,408
	21.09%	13.72%	31.33%	25.58%
*Sexual orientation*				
Heterosexual	1,992	2,133	3,306	2,822
	90.96%	86.01%	84.51%	66.95%
Sexual minority	198	347	606	1,393
	9.04%	13.99%	15.49%	33.05%
*Child ethnicity*				
White	4,323	4,218	4,550	4,617
	95.62%	95.99%	83.33%	83.58%
Ethnic minority	198	176	910	907
	4.38%	4.01%	16.67%	16.42%
*Weight status*				
Low to average weight	2,897	2,748	3,160	3,025
	83.27%	77.43%	66.18%	63.36%
Higher weight	582	801	1,615	1,749
	16.73%	22.57%	33.82%	36.64%
*Deprivation*				
Lower levels of deprivation	3,664	3,511	2,821	2,784
	92.76%	91.77%	60.19%	57.98%
More deprived	286	315	1,866	2,018
	7.24%	8.23%	39.81%	42.02%
*Rurality*				
Urban	n/a	n/a	3,551	3,618
			63.98%	64.52%
Rural	n/a	n/a	1,999	1,990
			36.02%	35.49%
Body dissatisfaction age 10/11 (M, SD)	0.20	0.29	2.34	2.63
	(0.40)	(0.45)	(1.65)	(1.65)
Body dissatisfaction age 14 (M, SD)	23.17	27.92	2.74	3.65
	(8.47)	(9.36)	(1.38)	(1.62)

Total numbers in rows differ, based on the availability of non-missing data per variable

### Bullying in sexual minority youth

#### ALSPAC

Being bullied in childhood was reported by 805 (19.5%) heterosexual youth and 123 (22.6%) sexual minority youth, which was not significantly different. Being bullied in childhood was significantly positively related to body dissatisfaction for heterosexual girls at age 10 (B = .37 [.09; .64], *p* = .009) and age 14 (B = 1.47 [.19; 2.75, *p* = .024). No other significant associations emerged, and no moderation was found.

#### MCS

Being bullied in childhood was reported by 1,647 (26.9%) of heterosexual youth and 633 (31.7%) sexual minority youth, which was significantly different with a small effect size (*X*^*2*^(1) = 17.12, *p* < .001, *V* = .05). While no significant moderation effect was identified, being bullied in childhood was significantly positively related to body dissatisfaction for heterosexual boys at age 11 (β = .32 [.21; .44], *p* < .001), and age 14 (β = .24 [.14; .35], *p* < .001), as well as heterosexual girls at age 11 (β = .41 [.27; .54], *p* < .001) and age 14 (β = .22 [.10; .34], *p* < .001). Significant effects were also found for sexual minority boys at age 11 (β = .32 [.009; .64], *p* = .04) and 14 (β = .28 [.08; .48], *p* = .006), as well as sexual minority girls at age 11 (β = .38 [.21; .55], *p* < .001). No other significant associations emerged.

### Bullying in ethnic minority youth

#### ALSPAC

Being bullied in childhood was reported by 1,509 (17.7%) White youth and 71 (19.0%) ethnic minority youth, which was not significantly different. Being bullied in childhood was significantly positively related to body dissatisfaction for White girls at age 10 (B = .39 [.13; .65], *p* = .003) and age 14 (B = 1.34 [.13; 2.55], *p* = .029). No other significant associations emerged, and no moderation was found.

#### MCS

Being bullied in childhood was reported by 2,556 (27.9%) White youth and 592 (32.6%) ethnic minority youth, which was significantly different with a small effect size (*X*^*2*^(1) = 16.13, *p* < .001, *V* = .04). The only significant interaction term identified was for girls at age 14 (β = − .38 [ − .64;  − .12], *p* = .004), indicating a moderation effect (i.e., a weaker effect for ethnic minority girls). Being bullied in childhood was significantly positively related to body dissatisfaction for White boys at age 11 (β = .36 [.25; .48], *p* < .001) and age 14 (β = .24 [.14; .34], *p* < .001), as well as White girls at age 11 (β = .44 [.32; .55], *p* < .001) and age 14 (β = .24 [.14; .34], *p* < .001). Significant effects were also found for ethnic minority girls at age 11 (β = .26 [.01; .52], *p* = .04) and ethnic minority boys at age 14 (β = .28 [.04; .52], *p* = .02). No other significant associations emerged.

### Bullying in weight minority youth

#### ALSPAC

Being bullied in childhood was reported by 1,128 (20.0%) low to average weight youth and 316 (22.9%) higher weight youth, which was significantly different with a small effect size (*X*^*2*^(1) = 5.67, *p* = .017, *V* = .03). Being bullied in childhood was significantly positively related to body dissatisfaction for low to average weight girls at age 10 (B = .43 [.14; .72], *p* = .003). No other significant associations emerged, and no moderation was found.

#### MCS

Being bullied in childhood was reported by 1,617 (26.1%) low to average weight youth and 1,131 (33.6%) higher weight youth, which was significantly different with a small effect size (*X*^*2*^(1) = 56.33, *p* < .001, *V* = .08). While no significant moderation effect was identified, being bullied in childhood was significantly positively related to body dissatisfaction in low to average weight boys at age 11 (β = .31 [.17; .45], *p* < .001) and age 14 (β = .26 [.13; .38, *p* < .001), as well as low to average weight girls at age 11 (β = .48 [.34; .62], *p* < .001) and age 14 (β = .22 [.09; .34], *p* = .001). Significant effects were also identified for higher weight boys at age 11 (β = .33 [.12; .52], *p* = .001) and age 14 (β = .24 [.10; .39], *p* = .001), as well as higher weight girls at age 11 (β = .29 [.14; .44], *p* < .001). No other significant associations emerged.

### Bullying in deprived youth

#### ALSPAC

Childhood bullying was reported by 1,351 (18.8%) less deprived youth and 122 (20.3%) most deprived youth, which was not significantly different. Being bullied in childhood was significantly positively related to body dissatisfaction for less deprived boys at age 10 (B = .27 [.02; .52], *p* = .031), and less deprived girls at age 10 (B = .36 [.10; .62], *p* = .007) and age 14 (B = 1.51 [.28; 2.74], *p* = .016). No other significant associations emerged, and no moderation was found.

#### MCS

Being bullied in childhood was reported by 1,364 (24.3%) less deprived youth and 1,351 (34.8%) most deprived youth, which was significantly different with a small effect size (*X*^*2*^(1) = 122.62, *p* < .001, *V* = .11). While no significant moderation effect was identified, being bullied in childhood was significantly positively related to body dissatisfaction for less-deprived boys at age 11 (β = .34 [.23; .45], *p* < .001) and age 14 (β = .26 [.14; .38], *p* < .001), and less-deprived girls at age 11 (β = .48 [.33; .63], *p* < .001) and age 14 (β = .21 [.11; .32], *p* < .001). Significant effects were also identified for deprived boys at age 11 (β = .30 [.10; .49], *p* = .003) and age 14 (β = .23 [.08; .38], *p* = .003), as well as deprived girls at age 11 (β = .30 [.15; .45], *p* < .001). No other significant associations emerged.

### Bullying in rural and urban youth

#### MCS

Being bullied in childhood was reported by 2,131 (29.7%) urban youth and 1,191 (29.9%) rural youth, which was not significantly different. While no significant moderation effect was identified, being bullied in childhood was significantly positively related to body dissatisfaction for urban boys at age 11 (β = .29 [.15; .42], *p* < .001) and age 14 (β = .25 [.15; .36], *p* < .001), and urban girls at age 11 (β = .37 [.26; .49]) and age 14 (β = .21 [.11; .31], *p* < .001). Significant effects were also identified for rural boys at age 11 (β = .43 [.25; .60], *p* < .001) and age 14 (β = .25 [.07; .43], *p* = .008), and rural girls at age 11 (β = .48 [.26; .71], *p* < .001), *p* < .001). No other significant associations emerged.

## Discussion

The impact of being bullied in childhood on adolescent body dissatisfaction, especially marginalised youth, is an increasing area of interest. This study contributes valuable insights to the growing body of research by examining the relationship between childhood bullying and adolescent body dissatisfaction in different marginalised groups across two different cohorts. Collectively, our findings support the notion that bullying victimisation is a potential risk factor for body image disturbance [1]. In regard to marginalised groups, present findings did not provide evidence for the included groups to be at a higher risk for childhood bullying experiences affecting body image outcomes, despite higher levels of bullying victimisation among certain minority groups (sexual minorities, ethnic minorities, and more deprived adolescents in the MCS, and higher weight adolescents in both cohorts). These findings suggest that while general bullying victimisation may be a contributing factor to body dissatisfaction in youth from marginalised groups, there is a clear need to collect further information on the content and severity of bullying, to include larger populations of marginalised youth, and to develop and test aetiological risk models that consider cultural, ethnic, and weight-based diversity.

For example, previous research which specifically investigated the role of identity-based bullying on racial-ethnic minorities’ health outcomes found that young people exposed to bullying related to their race/ethnicity/national origin displayed increased mental health risks compared to adolescents who did not report any such experiences [8]. Moreover, specifically appearance-related bullying might have a unique impact on body image experiences, such as body shame and body dissatisfaction [39], and bullying experiences that target physical characteristics linked to marginalised identities, such as higher weight status or minority ethnic groups, might further uniquely impact body image [40].

It is important to note that findings from the current study need to be interpreted with care, as potentially influential factors might not have been captured in the present cohort datasets (e.g., differences between marginalised subgroups, types of bullying experiences) and analyses (e.g., the effect of intersectionality) due to study design and available sample size. Considering the wide confidence intervals identified, especially within marginalised groups, small sample sizes and varying experiences within groups likely impacted current findings. Additionally, as more specific information on bullying experiences and body image concerns were not available, future research will need to address the role of cultural factors on both bullying and body image experiences under consideration of intersecting identities.

Interestingly, findings for 14-year-old marginalised girls show a unique pattern of association in the MCS cohort, considering that a significant moderation effect was found for ethnicity in this subgroup of the MCS (i.e., a weaker effect of childhood bullying on ethnic minority girls’ body satisfaction). As puberty and associated physiological changes are associated with a higher risk for body image concerns in girls of this age [41], and body image dissatisfaction may vary between different marginalised ethnic groups [19], further research is needed to explore potential within-group differences.

While the current study had several strengths, including the longitudinal design and inclusion of two distinct cohort studies, several limitations should be noted. Firstly, all studies collected data on childhood bullying around the late 1990s and early 2000s. While the impacts of bullying are likely similar across decades, the nature of bullying has changed over time with decreases in physical forms of bullying and increases in digital forms of bullying [42, 43]. Secondly, due to study attrition over time we encountered high levels of missing data. To counter this issue, we tried to minimise biases in attrition by using multiple imputation accounting for auxiliary variables linked to missingness. Further, bullying experiences in both cohorts were assessed only regarding general bullying experiences. Future research will need to investigate how bullying specifically targeting weight, shape, and appearance might impact later body image concerns. Thirdly, the present analyses only investigated effects related to participants’ reported sex (as assigned at birth), while non-binary and changing gender identities could not be assessed due to the respective cohort study design. Considering that gender experiences (e.g., gender roles, expression, objectification) have been found to play a central role in mental health outcomes across time (e.g. [44]), future research will need to study how different or changing gender identities impact the relationship between bullying and body image experiences. Lastly, while we focused on a range of marginalised groups, we were unable to consider the intersectionality between these groups in the current study.

## Conclusions

Based on data from two UK-based cohort studies, experiences of being bullied in childhood can be considered a risk factor for later body image concerns. While the current study could not identify higher risks for this association in marginalised groups potentially due to sample size and distribution, the present findings suggest a need for more intersectional research investigating the role of multiple marginalised identities. Future studies will need to be large enough and use more nuanced and culturally sensitive assessment methods.

## Supplementary Information

Below is the link to the electronic supplementary material.

Supplementary Material 1

## Data Availability

This is a secondary-data analysis. Please note that the study website of the ALSPAC cohort contains details of all the data that is available through a fully searchable data dictionary and variable search tool: [https://www.bristol.ac.uk/alspac/researchers/our-data/](https:/www.bristol.ac.uk/alspac/researchers/our-data) . Data for the Millennium Cohort Study is available through the UK data service: [https://ukdataservice.ac.uk/](https:/ukdataservice.ac.uk) .

## Abbreviations

ALSPAC: Avon Longitudinal Study of Parents and Children
MCS: Millennium Cohort Study

## References

[1] Day S, Bussey K, Trompeter N, Mitchison D. The impact of teasing and bullying victimization on disordered eating and body image disturbance among adolescents: a systematic review. Trauma Violence Abuse. 2022;23(3):985–1006. 10.1177/1524838020985534 33461439

[2] Lereya ST, Copeland WE, Costello EJ, Wolke D. Adult mental health consequences of peer bullying and maltreatment in childhood: two cohorts in two countries. Lancet Psychiatry. 2015;2(6):524–31. 10.1016/S2215-0366(15)00165-0 PMC4580734 26360448

[3] Mitchison D, Bussey K, Touyz S, Gonzalez-Chica D, Musker M, Stocks N, et al. Shared associations between histories of victimisation among people with eating disorder symptoms and higher weight. Australian New Z J Psychiatry. 2019;53(6):540–9. 10.1177/0004867418814961 30501408

[4] Olweus D. Bully/victim problems among schoolchildren: Basic facts and effects of a school based intervention program. The development and treatment of childhood aggression: Psychology; 2013. pp. 411–48.

[5] Hymel S, Swearer SM. Four decades of research on school bullying: An introduction. Am Psychol. 2015;70(4):293. 10.1037/a0038928 25961310

[6] Hosozawa M, Bann D, Fink E, Elsden E, Baba S, Iso H, et al. Bullying victimisation in adolescence: prevalence and inequalities by gender, socioeconomic status and academic performance across 71 countries. eClinicalMedicine. 2021;41:101142. 10.1016/j.eclinm.2021.101142 PMC8517283 34693231

[7] Lawrence SE, McMorris BJ, Simon KA, Gower AL, Eisenberg ME. Bullying Involvement at the Intersection of Gender Identity/Modality, Sexual Identity, Race, Ethnicity, and Disability: Prevalence Disparities and the Role of School-Related Developmental Assets. LGBT Health. 2023;10(1suppl):S10–9. 10.1089/lgbt.2023.0076 PMC10623467 37754921

[8] Galán CA, Stokes LR, Szoko N, Abebe KZ, Culyba AJ. Exploration of Experiences and Perpetration of Identity-Based Bullying Among Adolescents by Race/Ethnicity and Other Marginalized Identities. JAMA Netw Open. 2021;4(7):e2116364–e. 10.1001/jamanetworkopen.2021.16364 PMC8303093 34297076

[9] Østergaard J, Pless M, Blackman S, MacDonald R. Introduction to special issue: youth, rural places and marginalisation. J Youth Stud. 2024;27(9):1227–39.

[10] Xu M, Macrynikola N, Waseem M, Miranda R. Racial and ethnic differences in bullying: Review and implications for intervention. Aggress Violent Beh. 2020;50:101340. 10.1016/j.avb.2019.101340 PMC7453877 32863731

[11] Potter LN, Brondolo E, Smyth JM. Biopsychosocial correlates of discrimination in daily life: A review. Stigma Health. 2019;4(1):38. 10.1037/sah0000120 PMC7286570 32524031

[12] Song C, Zhang Z, Wei K, Xie H. Divergent school bullying experiences and mental health: The mediating role of self-stigma among Chinese LGB adolescents and young adults. Child Abuse Negl. 2025;161:107306. 10.1016/j.chiabu.2025.107306 39914100

[13] Festinger L. A theory of social comparison processes. Hum Relat. 1954;7(2):117–40.

[14] Myers TA, Crowther JH. Social comparison as a predictor of body dissatisfaction: A meta-analytic review. J Abnorm Psychol. 2009;118(4):683. 10.1037/a0016763 19899839

[15] Thornberg R. School bullying and fitting into the peer landscape: a grounded theory field study. Br J Sociol Educ. 2018;39(1):144–58.

[16] Gleason JH, Alexander AM, Somers CL. Later adolescents’ reactions to three, types of childhood teasing: relations with self-esteem and body image. Soc Behav Pers Int J. 2000;28(5):471–9.

[17] Longitudinal associations among. bullying victimization, self-esteem, and adolescents’ depressive symptoms [press release]. US: Educational Publishing Foundation; 2024.

[18] Gardella JH, Fisher BW, Teurbe-Tolon AR, Ketner B, Nation M. Students’ Reasons for Why They Were Targeted for In-School Victimization and Bullying. Int J Bullying Prev. 2020;2(2):114–28.

[19] Kimber M, Couturier J, Georgiades K, Wahoush O, Jack SM. Ethnic Minority Status and Body Image Dissatisfaction: A Scoping Review of the Child and Adolescent Literature. J Immigr Minor Health. 2015;17(5):1567–79. 10.1007/s10903-014-0082-z 25115290

[20] He J, Sun S, Lin Z, Fan X. Body dissatisfaction and sexual orientations: A quantitative synthesis of 30 years research findings. Clin Psychol Rev. 2020;81:101896. 10.1016/j.cpr.2020.101896 32835956

[21] Nowicki GP, Marchwinski BR, O’Flynn JL, Griffths S, Rodgers RF. Body image and associated factors among sexual minority men: A systematic review. Body Image. 2022;43:154–69. 10.1016/j.bodyim.2022.08.006 36150358

[22] Esposito L, Villaseñor A. Relative deprivation: Measurement issues and predictive role for body image dissatisfaction. Soc Sci Med. 2017;192:49–57. 10.1016/j.socscimed.2017.09.029 28961482

[23] Hahn JS, Flouri E, Harrison A, Lewis G, Solmi F. Family Socioeconomic Position and Eating Disorder Symptoms Across Adolescence. JAMA Netw Open. 2025;8(8):e2527934–e. 10.1001/jamanetworkopen.2025.27934 PMC12368680 40833695

[24] Swami V, Tovée MJ. Female physical attractiveness in Britain and Malaysia: A cross-cultural study. Body Image. 2005;2(2):115–28. 10.1016/j.bodyim.2005.02.002 18089180

[25] Gaffney H, Ttofi MM, Farrington DP. Effectiveness of school-based programs to reduce bullying perpetration and victimization: An updated systematic review and meta-analysis. Campbell Syst Reviews. 2021;17(2):e1143. 10.1002/cl2.1143 PMC8356322 37131921

[26] Boyd A, Golding J, Macleod J, Lawlor DA, Fraser A, Henderson J, et al. Cohort Profile: The ‘Children of the 90s’—the index offspring of the Avon Longitudinal Study of Parents and Children. Int J Epidemiol. 2012;42(1):111–27. 10.1093/ije/dys064 PMC3600618 22507743

[27] Fraser A, Macdonald-Wallis C, Tilling K, Boyd A, Golding J, Davey Smith G, et al. Cohort Profile: The Avon Longitudinal Study of Parents and Children: ALSPAC mothers cohort. Int J Epidemiol. 2012;42(1):97–110. 10.1093/ije/dys066 PMC3600619 22507742

[28] London Uo. UCL Social Research Institute. Centre for Longitudinal Studies. Millennium Cohort Study; 2023.

[29] Shepherd P, Gilbert E. Millennium Cohort Study: Ethical Review and Consent. 2019.

[30] Houtepen, L. C., Heron, J., Suderman, M. J., Tilling, K., & Howe, L. D. (2018). Adverse childhood experiences in the children of the Avon Longitudinal Study of Parents and Children (ALSPAC). *Wellcome open research*, *3*, 106. https://doi.org/10.12688/wellcomeopenres.14716.1Houtepen L, Heron J, Suderman M, Tilling K, Howe L. Adverse childhood experiences in the children of t he Avon Longitudinal Study of Parents and Children (ALSPAC) [version 1; peer review: 3 approved]. Wellcome Open Res. 2018;3(106). 10.12688/wellcomeopenres.14716.1 PMC6281007 30569020

[31] Brownell KD, Kelman JH, Stunkard AJ. Treatment of obese children with and without their mothers: changes in weight and blood pressure. Pediatrics. 1983;71(4):515–23. 6835735

[32] Stice E. A prospective test of the dual-pathway model of bulimic pathology: Mediating effects of dieting and negative affect. J Abnorm Psychol. 2001;110(1):124–35. 10.1037//0021-843x.110.1.124 11261386

[33] Opitz M-C, Savage N, Talbot K, Trompeter N, Moody S, Micali N, et al. Is one item enough? Testing the potential utility of single-item assessments of appearance satisfaction across three studies. J Eat Disorders. 2025;13(1):296. 10.1186/s40337-025-01468-8 PMC12750883 41276855

[34] Cole TJ, Bellizzi MC, Flegal KM, Dietz WH. Establishing a standard definition for child overweight and obesity worldwide: international survey. BMJ. 2000;320(7244):1240. 10.1136/bmj.320.7244.1240 PMC27365 10797032

[35] Cole TJ, Freeman JV, Preece MA. Body mass index reference curves for the UK, 1990. Arch Dis Child. 1995;73(1):25–9. 10.1136/adc.73.1.25 PMC1511150 7639544

[36] Townsend P, Phillimore P, Beattie A. Health and deprivation: inequality and the North. Routledge; 2023.

[37] Staatz CB, Kelly Y, Lacey RE, Hardy R. Area-level and family-level socioeconomic position and body composition trajectories: longitudinal analysis of the UK Millennium Cohort Study. Lancet Public Health. 2021;6(8):e598–607. 10.1016/S2468-2667(21)00134-1 PMC8342403 34332672

[38] Madley-Dowd P, Hughes R, Tilling K, Heron J. The proportion of missing data should not be used to guide decisions on multiple imputation. J Clin Epidemiol. 2019;110:63–73. 10.1016/j.jclinepi.2019.02.016 PMC6547017 30878639

[39] Prince T, Mulgrew KE, Driver C, Mills L, Loza J, Hermens DF. Appearance-related cyberbullying and its association with the desire to alter physical appearance among adolescent females. J Eat Disorders. 2024;12(1):125. 10.1186/s40337-024-01083-z PMC11365200 39215341

[40] Kahle L, Peguero AA, Bodies, Bullying. The Interaction of Gender, Race, Ethnicity, Weight, and Inequality With School Victimization. Victims Offenders. 2017;12(2):323–45.

[41] Forney KJ, Keel PK, O’Connor S, Sisk C, Burt SA, Klump KL. Interaction of hormonal and social environments in understanding body image concerns in adolescent girls. J Psychiatr Res. 2019;109:178–84. 10.1016/j.jpsychires.2018.12.008 PMC6317862 30553150

[42] Kennedy RS. Bullying Trends in the United States: A Meta-Regression. Trauma Violence Abuse. 2021;22(4):914–27. 10.1177/1524838019888555 31789118

[43] Trompeter N, Jackson E, Sheanoda V, Luo A, Allison K, Bussey K. Cyberbullying prevalence in Australian adolescents: Time trends 2015–2020. J School Violence. 2022;21(3):252–65.

[44] Lereya ST, Norton S, Crease M, Deighton J, Labno A, Ravaccia GG, et al. Gender, marginalised groups, and young people’s mental health: a longitudinal analysis of trajectories. Child Adolesc Psychiatry Mental Health. 2024;18(1):29. 10.1186/s13034-024-00720-4 PMC10902968 38419124

