# Supplementary material for: Childhood bullying and body dissatisfaction in adolescents from marginalised groups
Source: J Eat Disord. 2026 Jul 22;14:244. doi: 10.1186/s40337-026-01709-4 (PMC13679007; doi:10.1186/s40337-026-01709-4)
Supplement: Supplementary file 1 [file 40337_2026_1709_MOESM1_ESM.docx]

**Childhood bullying and body dissatisfaction in adolescents from marginalised groups**

**Author details:**

*Marie-Christine Opitz	Department of Clinical Psychology, School of Health in Social Sciences, University of Edinburgh, Edinburgh, UK [joint first author]

Nora Trompeter	Great Ormond Street Institute of Child Health, UCL, London, UK [joint first author]

Ulrike Schmidt	Department of Psychological Medicine, King's College London, Institute of Psychiatry, Psychology and Neuroscience, London, UK

Nadia Micali	Great Ormond Street Institute of Child Health, UCL, London, UK; Center for Eating and feeding Disorders Research, Mental Health Center Ballerup, Copenhagen University Hospital – Mental Health Services CPH, Denmark [joint senior author]

Helen Sharpe	Department of Clinical Psychology, School of Health in Social Sciences, University of Edinburgh, Edinburgh, UK [joint senior author]

*Corresponding author (mopitz@ed.ac.uk)

**Supplementary Material A: All Regression Results ALSPAC Cohort**

|  | B | SE | *t* | *p* |
| --- | --- | --- | --- | --- |
| Bullying (yes) | 0.54 | 0.35 | 1.52 | .119 |
| Ethnicity (minority status) | 0.52 | 0.77 | 0.68 | .497 |
| Deprivation (most deprived) | 0.80 | 0.98 | 0.81 | .416 |
| Weight status (overweight/obese) | 1.19 | 0.40 | 3.01 | .003 |

**Boys – sexual minority age 10 outcome**

**Boys – heterosexual age 10 outcome**

|  | B | SE | *t* | *P* |
| --- | --- | --- | --- | --- |
| Bullying (yes) | 0.23 | 0.13 | 1.71 | .087 |
| Ethnicity (minority status) | 0.22 | 0.30 | 0.75 | .450 |
| Deprivation (most deprived) | 0.13 | 0.27 | 0.50 | .614 |
| Weight status (overweight/obese) | 1.45 | 0.15 | 9.70 | <.001 |

**Girls – heterosexual age 10 outcome**

|  | B | SE | *t* | *p* |
| --- | --- | --- | --- | --- |
| Bullying (yes) | 0.37 | 0.14 | 2.62 | .009 |
| Ethnicity (minority status) | 0.65 | 0.29 | 2.29 | .022 |
| Deprivation (most deprived) | -0.27 | 0.13 | -1.10 | .269 |
| Weight status (overweight/obese) | 1.60 | 0.13 | 12.63 | <.001 |

**Girls – sexual minority age 10 outcome**

|  | B | SE | *t* | *p* |
| --- | --- | --- | --- | --- |
| Bullying (yes) | 0.49 | 0.34 | 1.41 | .157 |
| Ethnicity (minority status) | -0.29 | 0.64 | -0.45 | .654 |
| Deprivation (most deprived) | -0.65 | 0.56 | -1.17 | .244 |
| Weight status (overweight/obese) | 1.12 | 0.30 | 3.74 | <.001 |

**Boys – heterosexual age 14 outcome**

|  | B | SE | *t* | *p* |
| --- | --- | --- | --- | --- |
| Bullying (yes) | 0.37 | 0.53 | 0.70 | .482 |
| Ethnicity (minority status) | -0.40 | 1.33 | -0.30 | .767 |
| Deprivation (most deprived) | -0.36 | 0.72 | -0.30 | .765 |
| Weight status (overweight/obese) | 3.93 | 0.72 | 5.44 | <.001 |

**Boys – sexual minority age 14 outcome**

|  | B | SE | *t* | *p* |
| --- | --- | --- | --- | --- |
| Bullying (yes) | 0.92 | 1.52 | 0.61 | .546 |
| Ethnicity (minority status) | -1.50 | 3.27 | -0.46 | .648 |
| Deprivation (most deprived) | -5.72 | 5.09 | -1.12 | .265 |
| Weight status (overweight/obese) | 8.24 | 1.75 | 4.70 | <.001 |

**Girls – heterosexual age 14 outcome**

|  | B | SE | *t* | *p* |
| --- | --- | --- | --- | --- |
| Bullying (yes) | 1.47 | 0.65 | 2.26 | .024 |
| Ethnicity (minority status) | 0.75 | 1.30 | 0.58 | .561 |
| Deprivation (most deprived) | -0.44 | 1.03 | -0.42 | .672 |
| Weight status (overweight/obese) | 4.20 | 0.57 | 7.35 | <.001 |

**Girls – sexual minority age 14 outcome**

|  | B | SE | *t* | *P* |
| --- | --- | --- | --- | --- |
| Bullying (yes) | -0.09 | 1.59 | -0.06 | .953 |
| Ethnicity (minority status) | 1.51 | 2.79 | 0.54 | .589 |
| Deprivation (most deprived) | 0.53 | 2.60 | 0.20 | .839 |
| Weight status (overweight/obese) | 2.72 | 1.43 | 1.90 | .059 |

**Boys – low to average weight status age 10 outcome**

|  | B | SE | *t* | *P* |
| --- | --- | --- | --- | --- |
| Bullying (yes) | 0.22 | 0.14 | 1.57 | .115 |
| Ethnicity (minority status) | 0.54 | 0.31 | 1.76 | .078 |
| Deprivation (most deprived) | -0.13 | 0.32 | -0.42 | .676 |
| Sexual orientation (sexual minority) | 0.50 | 0.20 | 2.45 | .014 |

**Boys – higher weight status age 10 outcome**

|  | B | SE | *t* | *P* |
| --- | --- | --- | --- | --- |
| Bullying (yes) | 0.44 | 0.28 | 1.56 | .120 |
| Ethnicity (minority status) | -0.68 | 0.57 | -1.20 | .229 |
| Deprivation (most deprived) | 1.23 | 0.58 | 2.09 | .036 |
| Sexual orientation (sexual minority) | 0.27 | 0.37 | 0.71 | .475 |

**Girls – low to average weight status age 10 outcome**

|  | B | SE | *t* | *P* |
| --- | --- | --- | --- | --- |
| Bullying (yes) | 0.43 | 0.15 | 2.93 | .003 |
| Ethnicity (minority status) | 0.61 | 0.29 | 2.10 | .035 |
| Deprivation (most deprived) | -0.58 | 0.29 | -2.04 | .042 |
| Sexual orientation (sexual minority) | 0.31 | 0.16 | 1.94 | .052 |

**Girls – higher weight status age 10 outcome**

|  | B | SE | *t* | *P* |
| --- | --- | --- | --- | --- |
| Bullying (yes) | 0.25 | 0.26 | 0.96 | .337 |
| Ethnicity (minority status) | -0.02 | 0.53 | -0.04 | .964 |
| Deprivation (most deprived) | 0.17 | 0.41 | 0.42 | .677 |
| Sexual orientation (sexual minority) | -0.18 | 0.28 | -0.63 | .531 |

**Boys – low to average weight status age 14 outcome**

|  | B | SE | *t* | *P* |
| --- | --- | --- | --- | --- |
| Bullying (yes) | 0.62 | 0.53 | 1.17 | .242 |
| Ethnicity (minority status) | -0.32 | 1.31 | -0.24 | .808 |
| Deprivation (most deprived) | -0.29 | 1.28 | -0.23 | .819 |
| Sexual orientation (sexual minority) | -0.23 | 0.86 | -0.27 | .787 |

**Boys – higher weight status age 14 outcome**

|  | B | SE | *t* | *P* |
| --- | --- | --- | --- | --- |
| Bullying (yes) | -0.58 | 1.48 | -0.39 | .694 |
| Ethnicity (minority status) | -1.24 | 3.01 | -0.41 | .680 |
| Deprivation (most deprived) | -2.05 | 2.69 | -0.76 | .447 |
| Sexual orientation (sexual minority) | 4.02 | 1.95 | 2.06 | .041 |

**Girls – low to average weight status age 14 outcome**

|  | B | SE | *t* | *P* |
| --- | --- | --- | --- | --- |
| Bullying (yes) | 1.07 | 0.69 | 1.56 | .119 |
| Ethnicity (minority status) | 0.73 | 1.34 | 0.54 | .587 |
| Deprivation (most deprived) | -0.69 | 1.06 | -0.65 | .518 |
| Sexual orientation (sexual minority) | 2.21 | 0.72 | 3.08 | .002 |

**Girls – higher weight status age 14 outcome**

|  | B | SE | *t* | *P* |
| --- | --- | --- | --- | --- |
| Bullying (yes) | 2.40 | 1.22 | 1.96 | .051 |
| Ethnicity (minority status) | 1.86 | 2.71 | 0.68 | .494 |
| Deprivation (most deprived) | 0.83 | 1.96 | 0.43 | .671 |
| Sexual orientation (sexual minority) | 0.40 | 1.34 | 0.30 | .766 |

**Boys – Less deprived age 10 outcome**

|  | B | SE | *t* | *P* |
| --- | --- | --- | --- | --- |
| Bullying (yes) | 0.27 | 0.13 | 2.15 | .031 |
| Ethnicity (minority status) | 0.22 | 0.30 | 0.71 | .476 |
| Sexual orientation (sexual minority) | 0.43 | 0.18 | 2.36 | .018 |
| Weight status (overweight/obese) | 1.35 | 0.14 | 9.41 | <.001 |

**Boys – More deprived age 10 outcome**

|  | B | SE | *t* | *P* |
| --- | --- | --- | --- | --- |
| Bullying (yes) | 0.18 | 0.69 | 0.26 | .797 |
| Ethnicity (minority status) | 0.37 | 0.73 | 0.50 | .618 |
| Sexual orientation (sexual minority) | 1.08 | 1.09 | 0.99 | .322 |
| Weight status (overweight/obese) | 2.41 | 0.63 | 3.83 | <.001 |

**Girls – Less deprived age 10 outcome**

|  | B | SE | *t* | *P* |
| --- | --- | --- | --- | --- |
| Bullying (yes) | 0.36 | 0.13 | 2.71 | .007 |
| Ethnicity (minority status) | 0.62 | 0.28 | 2.23 | .026 |
| Sexual orientation (sexual minority) | 0.22 | 0.15 | 1.52 | .128 |
| Weight status (overweight/obese) | 1.49 | 0.12 | 12.39 | <.001 |

**Girls – More deprived age 10 outcome**

|  | B | SE | *t* | *P* |
| --- | --- | --- | --- | --- |
| Bullying (yes) | 1.08 | 0.58 | 1.86 | .063 |
| Ethnicity (minority status) | -0.66 | 0.84 | -0.78 | .434 |
| Sexual orientation (sexual minority) | -0.29 | 0.64 | -0.45 | .652 |
| Weight status (overweight/obese) | 2.38 | 0.51 | 4.64 | <.001 |

**Boys – Less deprived age 14 outcome**

|  | B | SE | *t* | *P* |
| --- | --- | --- | --- | --- |
| Bullying (yes) | 0.44 | 0.51 | 0.86 | .390 |
| Ethnicity (minority status) | -0.75 | 1.31 | -0.57 | .566 |
| Sexual orientation (sexual minority) | 0.83 | 0.79 | 1.06 | .291 |
| Weight status (overweight/obese) | 4.59 | 0.66 | 6.90 | <.001 |

**Boys – More deprived age 14 outcome**

|  | B | SE | *t* | *P* |
| --- | --- | --- | --- | --- |
| Bullying (yes) | 0.34 | 2.82 | 0.12 | .905 |
| Ethnicity (minority status) | 0.50 | 3.32 | 0.15 | .881 |
| Sexual orientation (sexual minority) | -3.77 | 5.51 | -0.68 | .497 |
| Weight status (overweight/obese) | 2.80 | 2.83 | 0.99 | .326 |

**Girls – Less deprived age 14 outcome**

|  | B | SE | *t* | *P* |
| --- | --- | --- | --- | --- |
| Bullying (yes) | 1.51 | 0.63 | 2.41 | .016 |
| Ethnicity (minority status) | 1.03 | 1.28 | 0.80 | .421 |
| Sexual orientation (sexual minority) | 1.73 | 0.66 | 2.63 | .009 |
| Weight status (overweight/obese) | 3.82 | 0.55 | 6.94 | <.001 |

**Girls – More deprived age 14 outcome**

|  | B | SE | *t* | *P* |
| --- | --- | --- | --- | --- |
| Bullying (yes) | -2.04 | 2.36 | -0.86 | .391 |
| Ethnicity (minority status) | -0.17 | 3.28 | -0.05 | .958 |
| Sexual orientation (sexual minority) | 3.08 | 2.56 | 1.20 | .232 |
| Weight status (overweight/obese) | 5.63 | 2.07 | 2.72 | .008 |

**Boys – White age 10 outcome**

|  | B | SE | *t* | *P* |
| --- | --- | --- | --- | --- |
| Bullying (yes) | 0.22 | 0.13 | 1.76 | .078 |
| Weight status (overweight/obese) | 1.47 | 0.14 | 10.23 | <.001 |
| Deprivation (most deprived) | 0.13 | 0.28 | 0.48 | .633 |
| Sexual orientation (sexual minority) | 0.43 | 0.18 | 2.01 | .020 |

**Boys – ethnic minority age 10 outcome**

|  | B | SE | *t* | *P* |
| --- | --- | --- | --- | --- |
| Bullying (yes) | 1.11 | 0.60 | 1.85 | .063 |
| Weight status (overweight/obese) | 0.58 | 0.62 | 0.93 | .350 |
| Deprivation (most deprived) | 0.76 | 0.67 | 1.14 | .256 |
| Sexual orientation (sexual minority) | 0.83 | 0.79 | 1.04 | .297 |

**Girls – White age 10 outcome**

|  | B | SE | *t* | *P* |
| --- | --- | --- | --- | --- |
| Bullying (yes) | 0.39 | 0.13 | 2.98 | .003 |
| Weight status (overweight/obese) | 1.54 | 0.12 | 13.02 | <.001 |
| Deprivation (most deprived) | -0.24 | 0.23 | -1.06 | .288 |
| Sexual orientation (sexual minority) | 0.24 | 0.15 | 1.62 | .104 |

**Girls – ethnic minority age 10 outcome**

|  | B | SE | *t* | *P* |
| --- | --- | --- | --- | --- |
| Bullying (yes) | 0.19 | 0.65 | 0.29 | .770 |
| Weight status (overweight/obese) | 1.15 | 0.63 | 1.84 | .065 |
| Deprivation (most deprived) | -1.10 | 0.78 | -1.41 | .159 |
| Sexual orientation (sexual minority) | -0.67 | 0.68 | -0.98 | .326 |

**Boys – White age 14 outcome**

|  | B | SE | *t* | *P* |
| --- | --- | --- | --- | --- |
| Bullying (yes) | 0.56 | 0.52 | 1.08 | .282 |
| Weight status (overweight/obese) | 4.53 | 0.67 | 6.80 | <.001 |
| Deprivation (most deprived) | -0.77 | 1.29 | -0.60 | .359 |
| Sexual orientation (sexual minority) | 0.74 | 0.81 | 0.92 | .359 |

**Boys – ethnic minority age 14 outcome**

|  | B | SE | *t* | *P* |
| --- | --- | --- | --- | --- |
| Bullying (yes) | -2.81 | 2.60 | -1.08 | .287 |
| Weight status (overweight/obese) | 2.98 | 2.82 | 1.06 | .297 |
| Deprivation (most deprived) | -0.50 | 2.95 | -0.23 | .815 |
| Sexual orientation (sexual minority) | -0.80 | 3.39 | -0.23 | .815 |

**Girls – White age 14 outcome**

|  | B | SE | *t* | *P* |
| --- | --- | --- | --- | --- |
| Bullying (yes) | 1.34 | 0.61 | 2.18 | .029 |
| Weight status (overweight/obese) | 3.90 | 0.54 | 7.24 | <.001 |
| Deprivation (most deprived) | -0.22 | 1.00 | -0.22 | .824 |
| Sexual orientation (sexual minority) | 1.75 | 0.65 | 2.69 | .007 |

**Girls – ethnic minority age 14 outcome**

|  | B | SE | *t* | *P* |
| --- | --- | --- | --- | --- |
| Bullying (yes) | 0.77 | 3.14 | 0.25 | .807 |
| Weight status (overweight/obese) | 6.11 | 3.06 | 2.01 | .050 |
| Deprivation (most deprived) | -2.35 | 3.65 | -0.64 | .522 |
| Sexual orientation (sexual minority) | 3.24 | 3.65 | 1.07 | .292 |

**Supplementary Material B: All Regression Results Millennium Cohort Study**

**Boys – ethnic minority age 11 outcome**

|  | β | SE | t | P>\|t\| |
| --- | --- | --- | --- | --- |
| Bullying (yes) | 0.05 | 0.17 | 0.28 | 0.777 |
| Sexual orientation (sexual minority) | 0.79 | 0.40 | 2.01 | 0.046 |
| Weight status (higher weight) | 0.21 | 0.13 | 1.62 | 0.108 |
| Rurality (urban) | 0.34 | 0.21 | 1.67 | 0.096 |
| Deprivation (more deprived) |  |  |  |  |
| 2 | 0.07 | 0.31 | 0.21 | 0.835 |
| 3 | -0.03 | 0.21 | -0.13 | 0.901 |
| 4 | 0.01 | 0.21 | 0.05 | 0.963 |
| 5 | -0.29 | 0.20 | -1.48 | 0.142 |

**Boys – White age 11 outcome**

|  | β | SE | t | P>\|t\| |
| --- | --- | --- | --- | --- |
| Bullying (yes) | 0.36 | 0.06 | 6.19 | 0.000 |
| Sexual orientation (sexual minority) | 0.17 | 0.08 | 2.27 | 0.024 |
| Weight status (higher weight) | 0.19 | 0.05 | 3.74 | 0.000 |
| Rurality (urban) | 0.02 | 0.05 | 0.49 | 0.626 |
| Deprivation (more deprived) |  |  |  |  |
| 2 | -0.18 | 0.09 | -1.86 | 0.063 |
| 3 | -0.12 | 0.10 | -1.29 | 0.196 |
| 4 | -0.11 | 0.09 | -1.27 | 0.205 |
| 5 | -0.16 | 0.08 | -1.93 | 0.054 |

**Girls – ethnic minority age 11 outcome**

|  | β | SE | t | P>\|t\| |
| --- | --- | --- | --- | --- |
| Bullying (yes) | 0.26 | 0.13 | 2.07 | 0.041 |
| Sexual orientation (sexual minority) | 0.23 | 0.13 | 1.72 | 0.088 |
| Weight status (higher weight) | 0.12 | 0.12 | 0.98 | 0.326 |
| Rurality (urban) | -0.39 | 0.21 | -1.83 | 0.069 |
| Deprivation (more deprived) |  |  |  |  |
| 2 | 0.08 | 0.17 | 0.48 | 0.633 |
| 3 | 0.17 | 0.14 | 1.27 | 0.205 |
| 4 | 0.17 | 0.19 | 0.90 | 0.371 |
| 5 | 0.39 | 0.18 | 2.14 | 0.034 |

**Girls – White age 11 outcome**

|  | β | SE | t | P>\|t\| |
| --- | --- | --- | --- | --- |
| Bullying (yes) | 0.44 | 0.06 | 7.34 | 0.000 |
| Sexual orientation (sexual minority) | 0.11 | 0.05 | 2.36 | 0.019 |
| Weight status (higher weight) | 0.22 | 0.05 | 4.68 | 0.000 |
| Rurality (urban) | -0.02 | 0.04 | -0.49 | 0.623 |
| Deprivation (more deprived) |  |  |  |  |
| 2 | -0.03 | 0.09 | -0.35 | 0.730 |
| 3 | 0.01 | 0.09 | 0.13 | 0.897 |
| 4 | 0.05 | 0.08 | 0.68 | 0.498 |
| 5 | 0.01 | 0.08 | 0.17 | 0.864 |

**Boys – ethnic minority age 14 outcome**

|  | β | SE | t | P>\|t\| |
| --- | --- | --- | --- | --- |
| Bullying (yes) | 0.28 | 0.12 | 2.29 | 0.023 |
| Sexual orientation (sexual minority) | 0.06 | 0.13 | 0.49 | 0.627 |
| Weight status (higher weight) | 0.05 | 0.11 | 0.46 | 0.648 |
| Rurality (urban) | 0.33 | 0.25 | 1.29 | 0.198 |
| Deprivation (more deprived) |  |  |  |  |
| 2 | 0.07 | 0.17 | 0.41 | 0.683 |
| 3 | 0.17 | 0.12 | 1.42 | 0.156 |
| 4 | 0.13 | 0.15 | 0.92 | 0.360 |
| 5 | -0.02 | 0.13 | -0.13 | 0.897 |

**Boys – White age 14 outcome**

|  | β | SE | t | P>\|t\| |
| --- | --- | --- | --- | --- |
| Bullying (yes) | 0.24 | 0.05 | 4.65 | 0.000 |
| Sexual orientation (sexual minority) | 0.29 | 0.06 | 4.66 | 0.000 |
| Weight status (higher weight) | 0.27 | 0.05 | 5.66 | 0.000 |
| Rurality (urban) | 0.04 | 0.05 | 0.69 | 0.491 |
| Deprivation (more deprived) |  |  |  |  |
| 2 | -0.13 | 0.10 | -1.29 | 0.198 |
| 3 | -0.08 | 0.08 | -0.97 | 0.332 |
| 4 | -0.03 | 0.08 | -0.39 | 0.699 |
| 5 | -0.12 | 0.08 | -1.56 | 0.120 |

**Girls – ethnic minority age 14 outcome**

|  | β | SE | t | P>\|t\| |
| --- | --- | --- | --- | --- |
| Bullying (yes) | -0.07 | 0.11 | -0.62 | 0.534 |
| Sexual orientation (sexual minority) | 0.09 | 0.11 | 0.85 | 0.397 |
| Weight status (higher weight) | 0.22 | 0.11 | 1.93 | 0.055 |
| Rurality (urban) | -0.22 | 0.27 | -0.85 | 0.399 |
| Deprivation (more deprived) |  |  |  |  |
| 2 | 0.37 | 0.15 | 2.52 | 0.013 |
| 3 | 0.11 | 0.14 | 0.83 | 0.406 |
| 4 | 0.18 | 0.17 | 1.03 | 0.304 |
| 5 | 0.52 | 0.17 | 3.07 | 0.003 |

**Girls – White age 14 outcome**

|  | β | SE | t | P>\|t\| |
| --- | --- | --- | --- | --- |
| Bullying (yes) | 0.24 | 0.05 | 4.51 | 0.000 |
| Sexual orientation (sexual minority) | 0.29 | 0.04 | 6.91 | 0.000 |
| Weight status (higher weight) | 0.25 | 0.04 | 5.55 | 0.000 |
| Rurality (urban) | -0.06 | 0.05 | -1.18 | 0.237 |
| Deprivation (more deprived) |  |  |  |  |
| 2 | 0.06 | 0.08 | 0.70 | 0.482 |
| 3 | -0.04 | 0.07 | -0.57 | 0.571 |
| 4 | 0.004 | 0.07 | 0.06 | 0.949 |
| 5 | -0.09 | 0.07 | -1.27 | 0.205 |

**Boys – sexual minority age 11 outcome**

|  | β | SE | t | P>\|t\| |
| --- | --- | --- | --- | --- |
| Bullying (yes) | 0.32 | 0.16 | 2.02 | 0.044 |
| Ethnicity (ethnic minority) | 0.33 | 0.32 | 1.01 | 0.314 |
| Weight status (higher weight) | 0.09 | 0.12 | 0.75 | 0.452 |
| Rurality (urban) | -0.03 | 0.13 | -0.22 | 0.827 |
| Deprivation (more deprived) |  |  |  |  |
| 2 | -0.48 | 0.28 | -1.73 | 0.086 |
| 3 | -0.37 | 0.31 | -1.18 | 0.238 |
| 4 | -0.20 | 0.29 | -0.67 | 0.503 |
| 5 | -0.57 | 0.30 | -1.90 | 0.059 |

**Boys – heterosexual age 11 outcome**

|  | β | SE | t | P>\|t\| |
| --- | --- | --- | --- | --- |
| Bullying (yes) | 0.32 | 0.06 | 5.56 | 0.000 |
| Ethnicity (ethnic minority) | -0.12 | 0.08 | -1.39 | 0.167 |
| Weight status (higher weight) | 0.22 | 0.06 | 3.82 | 0.000 |
| Rurality (urban) | 0.06 | 0.05 | 1.22 | 0.222 |
| Deprivation (more deprived) |  |  |  |  |
| 2 | -0.05 | 0.12 | -0.46 | 0.644 |
| 3 | -0.05 | 0.08 | -0.67 | 0.506 |
| 4 | -0.06 | 0.08 | -0.76 | 0.450 |
| 5 | -0.09 | 0.07 | -1.30 | 0.195 |

**Girls – sexual minority age 11 outcome**

|  | β | SE | t | P>\|t\| |
| --- | --- | --- | --- | --- |
| Bullying (yes) | 0.38 | 0.09 | 4.33 | 0.000 |
| Ethnicity (ethnic minority) | -0.04 | 0.13 | -0.30 | 0.762 |
| Weight status (higher weight) | 0.11 | 0.08 | 1.43 | 0.154 |
| Rurality (urban) | -0.04 | 0.09 | -0.43 | 0.666 |
| Deprivation (more deprived) |  |  |  |  |
| 2 | -0.04 | 0.12 | -0.37 | 0.714 |
| 3 | -0.04 | 0.11 | -0.37 | 0.712 |
| 4 | -0.01 | 0.14 | -0.08 | 0.936 |
| 5 | 0.020 | 0.11 | 0.18 | 0.857 |

**Girls –heterosexual age 11 outcome**

|  | β | SE | t | P>\|t\| |
| --- | --- | --- | --- | --- |
| Bullying (yes) | 0.41 | 0.07 | 5.86 | 0.000 |
| Ethnicity (ethnic minority) | -0.14 | 0.08 | -1.90 | 0.058 |
| Weight status (higher weight) | 0.24 | 0.05 | 4.99 | 0.000 |
| Rurality (urban) | -0.05 | 0.05 | -0.99 | 0.323 |
| Deprivation (more deprived) |  |  |  |  |
| 2 | 0.03 | 0.10 | 0.28 | 0.782 |
| 3 | 0.10 | 0.10 | 1.00 | 0.319 |
| 4 | 0.12 | 0.08 | 1.64 | 0.103 |
| 5 | 0.09 | 0.09 | 1.00 | 0.317 |

**Boys –sexual minority age 14 outcome**

|  | β | SE | t | P>\|t\| |
| --- | --- | --- | --- | --- |
| Bullying (yes) | 0.28 | 0.10 | 2.75 | 0.006 |
| Ethnicity (ethnic minority) | -0.26 | 0.14 | -1.89 | 0.060 |
| Weight status (higher weight) | 0.22 | 0.12 | 1.87 | 0.062 |
| Rurality (urban) | 0.002 | 0.13 | 0.01 | 0.989 |
| Deprivation (more deprived) |  |  |  |  |
| 2 | -0.005 | 0.19 | -0.02 | 0.982 |
| 3 | 0.11 | 0.18 | 0.59 | 0.556 |
| 4 | 0.16 | 0.20 | 0.80 | 0.426 |
| 5 | 0.010 | 0.18 | 0.05 | 0.958 |

**Boys –heterosexual age 14 outcome**

|  | β | SE | t | P>\|t\| |
| --- | --- | --- | --- | --- |
| Bullying (yes) | 0.24 | 0.05 | 4.62 | 0.000 |
| Ethnicity (ethnic minority) | -0.11 | 0.07 | -1.64 | 0.101 |
| Weight status (higher weight) | 0.24 | 0.05 | 4.85 | 0.000 |
| Rurality (urban) | 0.06 | 0.06 | 1.02 | 0.306 |
| Deprivation (more deprived) |  |  |  |  |
| 2 | -0.10 | 0.10 | -0.99 | 0.323 |
| 3 | -0.06 | 0.08 | -0.69 | 0.491 |
| 4 | -0.010 | 0.07 | -0.14 | 0.888 |
| 5 | -0.10 | 0.07 | -1.45 | 0.147 |

**Girls –sexual minority age 14 outcome**

|  | β | SE | t | P>\|t\| |
| --- | --- | --- | --- | --- |
| Bullying (yes) | 0.09 | 0.09 | 1.07 | 0.285 |
| Ethnicity (ethnic minority) | -0.25 | 0.12 | -2.18 | 0.030 |
| Weight status (higher weight) | 0.25 | 0.06 | 3.99 | 0.000 |
| Rurality (urban) | -0.08 | 0.08 | -0.96 | 0.340 |
| Deprivation (more deprived) |  |  |  |  |
| 2 | 0.26 | 0.12 | 2.16 | 0.032 |
| 3 | 0.13 | 0.12 | 1.08 | 0.281 |
| 4 | 0.05 | 0.12 | 0.36 | 0.718 |
| 5 | 0.0006 | 0.11 | 0.01 | 0.996 |

**Girls –heterosexual age 14 outcome**

|  | β | SE | t | P>\|t\| |
| --- | --- | --- | --- | --- |
| Bullying (yes) | 0.22 | 0.06 | 3.61 | 0.000 |
| Ethnicity (ethnic minority) | -0.09 | 0.07 | -1.29 | 0.198 |
| Weight status (higher weight) | 0.22 | 0.06 | 3.90 | 0.000 |
| Rurality (urban) | -0.07 | 0.06 | -1.23 | 0.218 |
| Deprivation (more deprived) |  |  |  |  |
| 2 | 0.09 | 0.09 | 1.00 | 0.316 |
| 3 | -0.04 | 0.08 | -0.56 | 0.578 |
| 4 | 0.07 | 0.07 | 0.96 | 0.340 |
| 5 | 0.01 | 0.09 | 0.13 | 0.895 |

**Boys – higher weight status age 11 outcome**

|  | β | SE | t | P>\|t\| |
| --- | --- | --- | --- | --- |
| Bullying (yes) | 0.33 | 0.10 | 3.24 | 0.001 |
| Sexual orientation (sexual minority) | 0.15 | 0.11 | 1.41 | 0.158 |
| Ethnicity (ethnic minority) | -0.06 | 0.13 | -0.42 | 0.673 |
| Rurality (urban) | 0.08 | 0.08 | 0.89 | 0.375 |
| Deprivation (more deprived) |  |  |  |  |
| 2 | 0.06 | 0.17 | 0.36 | 0.716 |
| 3 | -0.009 | 0.12 | -0.07 | 0.940 |
| 4 | 0.05 | 0.11 | 0.47 | 0.636 |
| 5 | -0.002 | 0.12 | -0.01 | 0.989 |

**Boys – low to average weight status age 11 outcome**

|  | β | SE | t | P>\|t\| |
| --- | --- | --- | --- | --- |
| Bullying (yes) | 0.31 | 0.07 | 4.44 | 0.000 |
| Sexual orientation (sexual minority) | 0.34 | 0.12 | 2.72 | 0.007 |
| Ethnicity (ethnic minority) | -0.07 | 0.09 | -0.72 | 0.473 |
| Rurality (urban) | 0.03 | 0.06 | 0.49 | 0.627 |
| Deprivation (more deprived) |  |  |  |  |
| 2 | -0.27 | 0.12 | -2.19 | 0.029 |
| 3 | -0.19 | 0.11 | -1.64 | 0.102 |
| 4 | -0.19 | 0.12 | -1.65 | 0.101 |
| 5 | -0.29 | 0.11 | -2.59 | 0.010 |

**Girls – higher weight status age 11 outcome**

|  | β | SE | t | P>\|t\| |
| --- | --- | --- | --- | --- |
| Bullying (yes) | 0.29 | 0.08 | 3.83 | 0.000 |
| Sexual orientation (sexual minority) | 0.05 | 0.07 | 0.74 | 0.459 |
| Ethnicity (ethnic minority) | -0.19 | 0.11 | -1.67 | 0.097 |
| Rurality (urban) | -0.05 | 0.08 | -0.65 | 0.517 |
| Deprivation (more deprived) |  |  |  |  |
| 2 | 0.05 | 0.12 | 0.39 | 0.697 |
| 3 | -0.005 | 0.11 | -0.04 | 0.967 |
| 4 | 0.14 | 0.10 | 1.34 | 0.181 |
| 5 | 0.09 | 0.11 | 0.80 | 0.426 |

**Girls – low to average weight status age 11 outcome**

|  | β | SE | t | P>\|t\| |
| --- | --- | --- | --- | --- |
| Bullying (yes) | 0.48 | 0.07 | 6.65 | 0.000 |
| Sexual orientation (sexual minority) | 0.19 | 0.06 | 3.39 | 0.001 |
| Ethnicity (ethnic minority) | -0.06 | 0.08 | -0.74 | 0.459 |
| Rurality (urban) | -0.04 | 0.05 | -0.71 | 0.476 |
| Deprivation (more deprived) |  |  |  |  |
| 2 | -0.03 | 0.10 | -0.36 | 0.722 |
| 3 | 0.08 | 0.10 | 0.83 | 0.408 |
| 4 | 0.05 | 0.09 | 0.54 | 0.591 |
| 5 | 0.05 | 0.08 | 0.65 | 0.515 |

**Boys – higher weight status age 14 outcome**

|  | β | SE | t | P>\|t\| |
| --- | --- | --- | --- | --- |
| Bullying (yes) | 0.24 | 0.07 | 3.36 | 0.001 |
| Sexual orientation (sexual minority) | 0.24 | 0.10 | 2.57 | 0.010 |
| Ethnicity (ethnic minority) | -0.24 | 0.10 | -2.36 | 0.019 |
| Rurality (urban) | 0.04 | 0.09 | 0.43 | 0.665 |
| Deprivation (more deprived) |  |  |  |  |
| 2 | -0.17 | 0.12 | -1.37 | 0.172 |
| 3 | -0.06 | 0.10 | -0.67 | 0.500 |
| 4 | -0.10 | 0.10 | -0.97 | 0.331 |
| 5 | -0.13 | 0.09 | -1.38 | 0.169 |

**Boys – low to average weight status age 14 outcome**

|  | β | SE | t | P>\|t\| |
| --- | --- | --- | --- | --- |
| Bullying (yes) | 0.26 | 0.06 | 3.98 | 0.000 |
| Sexual orientation (sexual minority) | 0.27 | 0.08 | 3.28 | 0.001 |
| Ethnicity (ethnic minority) | -0.05 | 0.08 | -0.70 | 0.483 |
| Rurality (urban) | 0.06 | 0.07 | 0.92 | 0.358 |
| Deprivation (more deprived) |  |  |  |  |
| 2 | -0.002 | 0.11 | -0.02 | 0.983 |
| 3 | 0.01 | 0.10 | 0.12 | 0.907 |
| 4 | 0.10 | 0.10 | 1.08 | 0.281 |
| 5 | -0.04 | 0.09 | -0.48 | 0.633 |

**Girls – higher weight status age 14 outcome**

|  | β | SE | t | P>\|t\| |
| --- | --- | --- | --- | --- |
| Bullying (yes) | 0.12 | 0.08 | 1.55 | 0.122 |
| Sexual orientation (sexual minority) | 0.28 | 0.07 | 3.99 | 0.000 |
| Ethnicity (ethnic minority) | -0.20 | 0.12 | -1.68 | 0.094 |
| Rurality (urban) | -0.05 | 0.09 | -0.60 | 0.549 |
| Deprivation (more deprived) |  |  |  |  |
| 2 | 0.13 | 0.12 | 1.11 | 0.266 |
| 3 | -0.01 | 0.10 | -0.11 | 0.912 |
| 4 | -0.01 | 0.11 | -0.10 | 0.922 |
| 5 | -0.02 | 0.11 | -0.14 | 0.888 |

**Girls – low to average weight status age 14 outcome**

|  | β | SE | t | P>\|t\| |
| --- | --- | --- | --- | --- |
| Bullying (yes) | 0.22 | 0.07 | 3.31 | 0.001 |
| Sexual orientation (sexual minority) | 0.24 | 0.05 | 4.97 | 0.000 |
| Ethnicity (ethnic minority) | -0.10 | 0.07 | -1.43 | 0.154 |
| Rurality (urban) | -0.09 | 0.05 | -1.68 | 0.093 |
| Deprivation (more deprived) |  |  |  |  |
| 2 | 0.14 | 0.08 | 1.71 | 0.089 |
| 3 | 0.03 | 0.08 | 0.41 | 0.680 |
| 4 | 0.10 | 0.08 | 1.33 | 0.183 |
| 5 | 0.02 | 0.08 | 0.24 | 0.811 |

**Boys – rural age 11 outcome**

|  | β | SE | t | P>\|t\| |
| --- | --- | --- | --- | --- |
| Bullying (yes) | 0.43 | 0.09 | 4.81 | 0.000 |
| Sexual orientation (sexual minority) | 0.08 | 0.09 | 0.90 | 0.371 |
| Ethnicity (ethnic minority) | 0.20 | 0.21 | 0.95 | 0.344 |
| Weight status (higher weight) | 0.24 | 0.08 | 2.94 | 0.004 |
| Deprivation (more deprived) |  |  |  |  |
| 2 | -0.07 | 0.33 | -0.21 | 0.831 |
| 3 | -0.05 | 0.32 | -0.16 | 0.873 |
| 4 | -0.06 | 0.32 | -0.19 | 0.847 |
| 5 | -0.20 | 0.32 | -0.61 | 0.545 |

**Boys – urban age 11 outcome**

|  | β | SE | t | P>\|t\| |
| --- | --- | --- | --- | --- |
| Bullying (yes) | 0.29 | 0.07 | 4.17 | 0.000 |
| Sexual orientation (sexual minority) | 0.31 | 0.12 | 2.65 | 0.008 |
| Ethnicity (ethnic minority) | -0.07 | 0.09 | -0.83 | 0.405 |
| Weight status (higher weight) | 0.17 | 0.07 | 2.56 | 0.011 |
| Deprivation (more deprived) |  |  |  |  |
| 2 | -0.14 | 0.12 | -1.13 | 0.258 |
| 3 | -0.12 | 0.09 | -1.33 | 0.184 |
| 4 | -0.10 | 0.09 | -1.10 | 0.273 |
| 5 | -0.16 | 0.09 | -1.82 | 0.070 |

**Girls – rural age 11 outcome**

|  | β | SE | t | P>\|t\| |
| --- | --- | --- | --- | --- |
| Bullying (yes) | 0.48 | 0.12 | 4.20 | 0.000 |
| Sexual orientation (sexual minority) | 0.11 | 0.08 | 1.29 | 0.197 |
| Ethnicity (ethnic minority) | -0.42 | 0.23 | -1.91 | 0.057 |
| Weight status (higher weight) | 0.23 | 0.08 | 2.86 | 0.005 |
| Deprivation (more deprived) |  |  |  |  |
| 2 | -0.07 | 0.17 | -0.38 | 0.704 |
| 3 | 0.16 | 0.18 | 0.84 | 0.400 |
| 4 | 0.16 | 0.17 | 0.96 | 0.336 |
| 5 | 0.10 | 0.17 | 0.61 | 0.540 |

**Girls – urban age 11 outcome**

|  | β | SE | t | P>\|t\| |
| --- | --- | --- | --- | --- |
| Bullying (yes) | 0.37 | 0.06 | 6.47 | 0.000 |
| Sexual orientation (sexual minority) | 0.14 | 0.05 | 2.68 | 0.008 |
| Ethnicity (ethnic minority) | -0.08 | 0.07 | -1.23 | 0.219 |
| Weight status (higher weight) | 0.19 | 0.05 | 3.70 | 0.000 |
| Deprivation (more deprived) |  |  |  |  |
| 2 | 0.03 | 0.09 | 0.31 | 0.756 |
| 3 | 0.03 | 0.08 | 0.34 | 0.736 |
| 4 | 0.07 | 0.08 | 0.86 | 0.391 |
| 5 | 0.08 | 0.08 | 0.97 | 0.331 |

**Boys – rural age 14 outcome**

|  | β | SE | t | P>\|t\| |
| --- | --- | --- | --- | --- |
| Bullying (yes) | 0.25 | 0.09 | 2.66 | 0.008 |
| Sexual orientation (sexual minority) | 0.28 | 0.12 | 2.27 | 0.024 |
| Ethnicity (ethnic minority) | 0.15 | 0.27 | 0.56 | 0.577 |
| Weight status (higher weight) | 0.21 | 0.10 | 1.99 | 0.048 |
| Deprivation (more deprived) |  |  |  |  |
| 2 | -0.07 | 0.23 | -0.30 | 0.768 |
| 3 | -0.04 | 0.20 | -0.22 | 0.830 |
| 4 | -0.12 | 0.21 | -0.56 | 0.575 |
| 5 | -0.15 | 0.20 | -0.78 | 0.433 |

**Boys – urban age 14 outcome**

|  | β | SE | t | P>\|t\| |
| --- | --- | --- | --- | --- |
| Bullying (yes) | 0.25 | 0.05 | 4.71 | 0.000 |
| Sexual orientation (sexual minority) | 0.27 | 0.07 | 3.57 | 0.000 |
| Ethnicity (ethnic minority) | -0.16 | 0.07 | -2.31 | 0.022 |
| Weight status (higher weight) | 0.24 | 0.05 | 5.05 | 0.000 |
| Deprivation (more deprived) |  |  |  |  |
| 2 | -0.08 | 0.10 | -0.87 | 0.384 |
| 3 | -0.04 | 0.08 | -0.47 | 0.637 |
| 4 | 0.06 | 0.07 | 0.86 | 0.392 |
| 5 | -0.08 | 0.07 | -1.08 | 0.283 |

**Girls – rural age 14 outcome**

|  | β | SE | t | P>\|t\| |
| --- | --- | --- | --- | --- |
| Bullying (yes) | 0.03 | 0.10 | 0.31 | 0.758 |
| Sexual orientation (sexual minority) | 0.28 | 0.10 | 2.86 | 0.005 |
| Ethnicity (ethnic minority) | -0.21 | 0.32 | -0.68 | 0.500 |
| Weight status (higher weight) | 0.30 | 0.08 | 3.94 | 0.000 |
| Deprivation (more deprived) |  |  |  |  |
| 2 | -0.18 | 0.18 | -1.00 | 0.320 |
| 3 | -0.10 | 0.16 | -0.66 | 0.512 |
| 4 | -0.03 | 0.16 | -0.20 | 0.845 |
| 5 | -0.18 | 0.16 | -1.17 | 0.241 |

**Girls – rural age 14 outcome**

|  | β | SE | t | P>\|t\| |
| --- | --- | --- | --- | --- |
| Bullying (yes) | 0.21 | 0.05 | 3.98 | 0.000 |
| Sexual orientation (sexual minority) | 0.26 | 0.04 | 6.00 | 0.000 |
| Ethnicity (ethnic minority) | -0.13 | 0.06 | -2.01 | 0.045 |
| Weight status (higher weight) | 0.22 | 0.05 | 4.61 | 0.000 |
| Deprivation (more deprived) |  |  |  |  |
| 2 | 0.18 | 0.08 | 2.30 | 0.022 |
| 3 | 0.02 | 0.07 | 0.27 | 0.791 |
| 4 | 0.03 | 0.07 | 0.46 | 0.648 |
| 5 | 0.03 | 0.07 | 0.47 | 0.641 |

**Boys – more deprived age 11 outcome**

|  | β | SE | t | P>\|t\| |
| --- | --- | --- | --- | --- |
| Bullying (yes) | 0.30 | 0.10 | 2.97 | 0.003 |
| Sexual orientation (sexual minority) | 0.35 | 0.20 | 1.81 | 0.071 |
| Ethnicity (ethnic minority) | -0.02 | 0.13 | -0.11 | 0.911 |
| Weight status (higher weight) | 0.13 | 0.10 | 1.35 | 0.179 |
| Rurality (urban) | 0.05 | 0.11 | 0.46 | 0.645 |

**Boys – less deprived age 11 outcome**

|  | β | SE | t | P>\|t\| |
| --- | --- | --- | --- | --- |
| Bullying (yes) | 0.34 | 0.06 | 5.93 | 0.000 |
| Sexual orientation (sexual minority) | 0.20 | 0.08 | 2.60 | 0.010 |
| Ethnicity (ethnic minority) | -0.06 | 0.09 | -0.63 | 0.530 |
| Weight status (higher weight) | 0.22 | 0.06 | 3.92 | 0.000 |
| Rurality (urban) | 0.04 | 0.05 | 0.77 | 0.443 |
| Const | -0.18 | 0.03 | -5.34 | 0.000 |

**Girls – more deprived age 11 outcome**

|  | β | SE | t | P>\|t\| |
| --- | --- | --- | --- | --- |
| Bullying (yes) | 0.30 | 0.08 | 4.03 | 0.000 |
| Sexual orientation (sexual minority) | 0.17 | 0.07 | 2.42 | 0.016 |
| Ethnicity (ethnic minority) | -0.18 | 0.09 | -2.05 | 0.042 |
| Weight status (higher weight) | 0.18 | 0.07 | 2.73 | 0.007 |
| Rurality (urban) | -0.16 | 0.08 | -1.87 | 0.063 |
| Const | -0.12 | 0.06 | -2.19 | 0.029 |

**Girls – less deprived age 11 outcome**

|  | β | SE | t | P>\|t\| |
| --- | --- | --- | --- | --- |
| Bullying (yes) | 0.48 | 0.08 | 6.40 | 0.000 |
| Sexual orientation (sexual minority) | 0.11 | 0.06 | 1.93 | 0.055 |
| Ethnicity (ethnic minority) | -0.03 | 0.09 | -0.36 | 0.717 |
| Weight status (higher weight) | 0.20 | 0.05 | 3.99 | 0.000 |
| Rurality (urban) | -0.01 | 0.05 | -0.24 | 0.812 |
| Const | -0.17 | 0.04 | -3.99 | 0.000 |

**Boys – more deprived age 14 outcome**

|  | β | SE | t | P>\|t\| |
| --- | --- | --- | --- | --- |
| Bullying (yes) | 0.23 | 0.08 | 3.04 | 0.003 |
| Sexual orientation (sexual minority) | 0.20 | 0.10 | 1.92 | 0.056 |
| Ethnicity (ethnic minority) | -0.19 | 0.08 | -2.21 | 0.028 |
| Weight status (higher weight) | 0.25 | 0.07 | 3.47 | 0.001 |
| Rurality (urban) | 0.11 | 0.11 | 1.03 | 0.302 |
| Const | -0.12 | 0.06 | -1.94 | 0.054 |

**Boys – less deprived age 14 outcome**

|  | β | SE | t | P>\|t\| |
| --- | --- | --- | --- | --- |
| Bullying (yes) | 0.26 | 0.06 | 4.35 | 0.000 |
| Sexual orientation (sexual minority) | 0.31 | 0.07 | 4.54 | 0.000 |
| Ethnicity (ethnic minority) | -0.06 | 0.07 | -0.80 | 0.425 |
| Weight status (higher weight) | 0.24 | 0.05 | 4.65 | 0.000 |
| Rurality (urban) | 0.04 | 0.06 | 0.80 | 0.427 |
| Const | -0.20 | 0.03 | -5.92 | 0.000 |

**Girls – more deprived age 14 outcome**

|  | β | SE | t | P>\|t\| |
| --- | --- | --- | --- | --- |
| Bullying (yes) | 0.06 | 0.07 | 0.87 | 0.383 |
| Sexual orientation (sexual minority) | 0.27 | 0.07 | 3.95 | 0.000 |
| Ethnicity (ethnic minority) | -0.23 | 0.07 | -3.15 | 0.002 |
| Weight status (higher weight) | 0.25 | 0.07 | 3.77 | 0.000 |
| Rurality (urban) | -0.15 | 0.11 | -1.33 | 0.184 |
| Const | -0.07 | 0.06 | -1.18 | 0.237 |

**Girls – more deprived age 14 outcome**

|  | β | SE | t | P>\|t\| |
| --- | --- | --- | --- | --- |
| Bullying (yes) | 0.21 | 0.05 | 3.96 | 0.000 |
| Sexual orientation (sexual minority) | 0.26 | 0.04 | 6.02 | 0.000 |
| Ethnicity (ethnic minority) | -0.13 | 0.06 | -2.15 | 0.032 |
| Weight status (higher weight) | 0.23 | 0.05 | 4.64 | 0.000 |
| Rurality (urban) | 0 | (omitted) |  |  |
| Const | -0.17 | 0.04 | -4.81 | 0.000 |
